# Kinetic studies of *Candida parapsilosis* phagocytosis by macrophages and detection of intracellular survival mechanisms

**DOI:** 10.3389/fmicb.2014.00633

**Published:** 2014-11-20

**Authors:** Renáta Tóth, Adél Tóth, Csaba Papp, Ferenc Jankovics, Csaba Vágvölgyi, Maria F. Alonso, Judith M. Bain, Lars-Peter Erwig, Attila Gácser

**Affiliations:** ^1^Department of Microbiology, University of SzegedSzeged, Hungary; ^2^Institute of Genetics, Biological Research Centre of the Hungarian Academy of SciencesSzeged, Hungary; ^3^Aberdeen Fungal Group, Institute of Medical Sciences, University of AberdeenAberdeen, UK

**Keywords:** video microscopy, phagocytic stages, *Candida parapsilosis*, intracellular survival mechanisms, pseudohypha uptake

## Abstract

Even though the number of *Candida* infections due to non-albicans species like *C. parapsilosis* has been increasing, little is known about their pathomechanisms. Certain aspects of *C. parapsilosis* and host interactions have already been investigated; however we lack information about the innate cellular responses toward this species. The aim of our project was to dissect and compare the phagocytosis of *C. parapsilosis* to *C. albicans* and to another *Candida* species *C. glabrata* by murine and human macrophages by live cell video microscopy. We broke down the phagocytic process into three stages: macrophage migration, engulfment of fungal cells and host cell killing after the uptake. Our results showed increased macrophage migration toward *C. parapsilosis* and we observed differences during the engulfment processes when comparing the three species. The engulfment time of *C. parapsilosis* was comparable to that of *C. albicans* regardless of the pseudohypha length and spatial orientation relative to phagocytes, while the rate of host cell killing and the overall uptake regarding *C. parapsilosis* showed similarities mainly with *C. glabrata*. Furthermore, we observed difference between human and murine phagocytes in the uptake of *C. parapsilosis*. UV-treatment of fungal cells had varied effects on phagocytosis dependent upon which *Candida* strain was used. Besides statistical analysis, live cell imaging videos showed that this species similarly to the other two also has the ability to survive in host cells via the following mechanisms: yeast replication, and pseudohypha growth inside of phagocytes, exocytosis of fungal cells and also abortion of host cell mitosis following the uptake. According to our knowledge this is the first study that provides a thorough examination of *C. parapsilosis* phagocytosis and reports intracellular survival mechanisms associated with this species.

## Introduction

Although there is a wide range of opportunistic fungi that pose a threat to patients with impaired immunity, without question the most frequent cause of invasive opportunistic mycoses are *Candida* species (Pfaller and Diekema, 2007). *Candida albicans* is the leading causative agent responsible for serious fungal infections, however an epidemiological shift has occurred resulting in an increase in the prevalence of non-albicans *Candida* (NAC) species since the 1990s. Some reports suggest that *Candida glabrata* is the second most common *Candida* species responsible for invasive infections (Malani et al., 2005; Foster et al., 2007), whereas other studies place *Candida parapsilosis* in this position (Trofa et al., 2008; Hays et al., 2011). Differences between rates of infections caused by these species vary by geographical area and patient demographics (Malani et al., 2005; Chow et al., 2012; Guinea, 2014; Quindos, 2014). Globally the frequency of *C. albicans* has been decreasing, *C. glabrata* remains stable, while interestingly *C. parapsilosis* is rising. This might be due to the reduced susceptibility of the latter two species against certain antifungal agents, such as azoles and echinocandins (Chow et al., 2012; Guinea, 2014; Quindos, 2014). In addition *C. parapsilosis* is the predominant species responsible for invasive candidiasis in premature infants and is associated with neonatal mortality (Benjamin et al., 2004; Trofa et al., 2008; Chow et al., 2012; Quindos, 2014). Although all three species belong to the same genus, there are important differences in their genetics, cellular morphology, antifungal drug susceptibility and virulence. For example, the ability to undergo morphogenesis is a key factor for certain species to successfully invade the host. While *C. glabrata* only exists as a yeast, *C. albicans* is able to switch between yeast and hyphal forms and occasionally to pseudohyphae, *C. parapsilosis* is primarily a yeast or in pseudohyphae form (Trofa et al., 2008; Brunke and Hube, 2013).

It has long been known that phagocytic cells such as macrophages and neutrophils play a crucial role in innate immune responses during infection and either loss of these cells or their effector functions result in susceptibility (Brown, 2011). There are four distinct stages of the phagocytic process: (1) aggregation of phagocytes at the site of infection, (2) recognition of foreign agents via receptors, (3) ingestion of foreign particles, and (4) elimination of internalized particles through phagosome maturation and digestion with hydrolytic enzymes (Lewis et al., 2012a, 2013). Despite the wide range of anti-fungal strategies provided by macrophages, opportunistic fungal species have evolved survival mechanisms to evade these processes. It has been previously reported that *C. glabrata* and *C. albicans* cells are able to survive in macrophage phagosomes by inhibiting their maturation process, and both species can replicate inside macrophages after their ingestion (Benjamin et al., 2004; Seider et al., 2011; Vylkova and Lorenz, 2014). Moreover, secretion of hydrolytic enzymes and rapid hyphae formation provides an opportunity for *C. albicans* to escape from phagosomes (Brunke and Hube, 2013). Although *C. glabrata* lacks these abilities, it is able to survive over long periods in the host due to fungal autophagy, without triggering strong proinflammatory responses (Seider et al., 2011; Brunke and Hube, 2013). *C. parapsilosis* is known to have the ability to secrete certain hydrolases and form pseudohyphae enabling tissue penetration^[5]^, but its interaction with innate immune cells is less well studied. It has been shown that *C. parapsilosis* is efficiently phagocytosed and killed by macrophages and induces an inflammatory response (Nemeth et al., 2013, 2014; Toth et al., 2014). Certain aspects of adaptive immune responses have also been investigated (asymmetric T-helper cell responses along with reduced induction of Th17 responses of PBMCs in the presence of *C. parapsilosis* compared to *C. albicans*, Toth et al., 2013); however, little is known about the immune recognition of this pathogen as well as the intracellular events following its phagocytosis. Certain intracellular events after the uptake of *C. albicans* and *C. glabrata* have already been described suggesting intracellular survival of these species, however we lack information on how *C. parapsilosis* withstands the restricted environmental conditions following phagocytosis.

The distinct stages of *C. albicans* phagocytosis have already been investigated (Lewis et al., 2012a). The aim of the current study was to gain a better understanding on the interactions between the cellular components of the innate immune system and *C. parapsilosis*. We used live cell video microscopy to compare the temporal dynamics of phagocytosis of *C. albicans, C. glabrata*, and *C. parapsilosis* by murine and primary human macrophages.

## Materials and methods

### Preparation and staining of *Candida* strains

*C. albicans SC5314, C. glabrata* ATCC 2001, *C. parapsilosis* CLIB 214 and *C. parapsilosis* CLIB 214 *leu2*Δ*::FRT/leu2*Δ*::FRT::cprp10*Δ*::FRT-caRP10::TDH3p-caGFP/cpRP10* GFP-labeled strains were used. The strains were maintained at 4°C on YPD plates (1% yeast extract, 2% glucose, 2% peptone, and 2.5% agar). Before the experiments, *Candida* strains were grown overnight at 30°C in liquid YPD medium (1% yeast extract, 2% glucose, 2% peptone) with shaking at 200 rpm. The cells were washed three times with phosphate buffered saline (PBS), counted with a Neubauer chamber and diluted to the final concentration of 1 × 10^8^/ ml. To prepare UV-killed fungal strains, twenty doses of 20 mJ/cm^−2^ UV treatment was applied. One mg/ml FITC (Sigma, Dorset, UK) was used for the staining of yeast cells dissolved in 0.05 M carbonate-bicarbonate buffer (BDH Chemicals, VWR International Leicestershire, UK). Staining was performed in the dark for 10 min then cells were washed three times with PBS and suspended in 1 ml PBS. In order to generate a GFP-tagged *C. parapsilosis* strain, the pSFS2 vector (Reuss et al., 2004) was used to integrate the *C. albicans* SC5314 *RP10* locus to the *RP10* locus of the *C. parapsilosis* CLIB 214 *leu2*Δ/*leu2*Δ (Holland et al., 2014) strain. After this a *GFP* containing vector with a *TDH3* constitutive promoter (Barelle et al., 2004) and with the auxotrophic selection marker (*C. maltosa LEU2*, pSN40) was integrated to the *C. albicans RP10* locus. The *GFP* positivity of transformants was confirmed by fluorescent microscopy.

### Preparation of J774.1 mouse macrophage cell line

To maintain the J774.1 macrophages, Dulbecco's modified Eagle's medium (DMEM; Lonza, Slough, UK) was used supplemented with 2 mM L-glutamine (Invitrogen, Paisley, UK), 10% fetal calf serum (FCS; Biosera, Ringmer, UK) and 200 U/ml penicillin/streptomycin (Invitrogen, Paisley, UK). Cells were incubated at 37°C, in the presence of 5% CO_2_. For live video microscopy, 1, 2 × 10^5^ cell macrophages were plated on 8-well μ-slides (ibidi, Martinsried, Germany) in a volume of 300 μl and incubated overnight before the addition of *Candida* strains. Shortly before the experiments, the media was replaced with 300 μl pre-heated supplemented DMEM containing 1 μM LysoTracker Red DND-99 (Invitrogen, Paisley, UK). LysoTracker Red was used to label the acidic compartments of phagocytes.

### Preparation of human PBMC-derived macrophages

Human peripheral blood mononuclear cells (PBMCs) were isolated from the blood of healthy donors under approval from the University of Aberdeen's Institutional Review Board. The isolation and derivation of PBMCs followed a standard protocol with modifications as described previously in the work of Rudkin et al. (2013). PBMCs (7.5 × 10^5^ cell/ml) were plated on 8-well μ-slide (ibidi, Martinsried, Germany) in supplemented DMEM (Lonza, Slough, UK) and incubated at 37°C with 5% CO_2_ for 6 days. Immediately before the experiment, the media was replaced with 300 μl pre-heated supplemented DMEM containing 1 μM LysoTracker Red DND-99 (Invitrogen, Paisley, UK).

### Phagocytosis assay and live cell video microscopy

Phagocytosis assays were performed using a standardized protocol from the work of Lewis et al. (2012a, 2013). Live and UV treated *C. albicans* (SC5314), *C. glabrata* (ATCC 2001), and *C. parapsilosis* (CLIB 214) cells were added to Lysotracker red DND-99-stained (Invitrogen, Paisley, UK) (1 μM) 4 × 10^5^ /ml J774.1 murine and 7.5 × 10^5^ /ml human PBMC-derived macrophages using the ratio 3:1 on 8-well μ-slides (ibidi, Martinsried, Germany) in 300 μl volumes immediately before the experiment. The ratio of *C. parapsilosis* cells to macrophages was 3:1. an UltraVIEW VoX Spinning disk confocal microscope (PerkinElmer, Massachusetts, USA) was used for video microscopy in a chamber set at 37°C with 5% CO_2_. Images were taken over a 6 h period with a CCD camera. For live cell imaging 40× oil immersion objective with 1.3. NA of the spinning disk confocal microscope was used. Four independent experiments were performed in J774.1 macrophages for each strain and at least two videos were analyzed from each experiment. More than 100 macrophages were selected and followed individually at defined time intervals over a 6 h period. At least two independent experiments were carried out in human PBMC-derived macrophages for each strain and at least two videos were analyzed per experiment with more than fifty macrophages selected and followed individually. All macrophages present in the microscopic field of view were followed individually at defined time intervals over a 6 h period. Volocity 6.3 image analysis software (Improvision, PerkinElmer, Coventry, UK) was used for tracking and statistical analysis of macrophage migration. Measurements included the macrophage migration toward *Candida* cells, engulfment time, distribution of fungal cells per macrophages, average uptake of fungal cells by actively phagocytosing macrophages, prevalence of uptake events, post-ingestion rupture of macrophages and the pseudohyphae orientation and length of *C. parapsilosis* cells.

The calculation of the average velocity of macrophages in the presence of different *Candida* strains was achieved using high throughput analysis with Volocity software 6.3. The engulfment time is the time difference between the initiation of the macrophage-fungal cell contact and the end of the full engulfment with the macrophage membrane enclosing around the ingested cell. Distribution of fungal cells per macrophages is the percentage of macrophages taking up defined number of fungal cell and the average uptake stands for the mean number of fungal cells taken up by phagocytes that ingested at least one yeast cell over the 6 h period. The prevalence of uptake stands for the percentage of uptake events during specified time intervals. Individual events imply the starting point of the recognition of fungal cells by phagocytic cells. Post-ingestion macrophage rupture is defined as the percentage of dead macrophages relative to the macrophage population after the uptake of fungal cells. Individual post-ingestion rupture events were visible up until the end of the 6 h of co-incubation which we defined as the disruption of membrane integrity.

### Statistical analysis

Volocity software enabled high-throughput migration analysis and provided information on the velocity of individual macrophages. Data were used to calculate the mean track velocity of phagocytes cultured with *Candida* strains.

Unpaired, two-tailed *t*-tests were used to determine statistical significance by GraphPad Prism v 5.0 software. Significant differences were considered at *P*-values of ≤0.05.

## Results

### Differences in macrophage phagocytosis between *Candida* species

**Figure 1:**
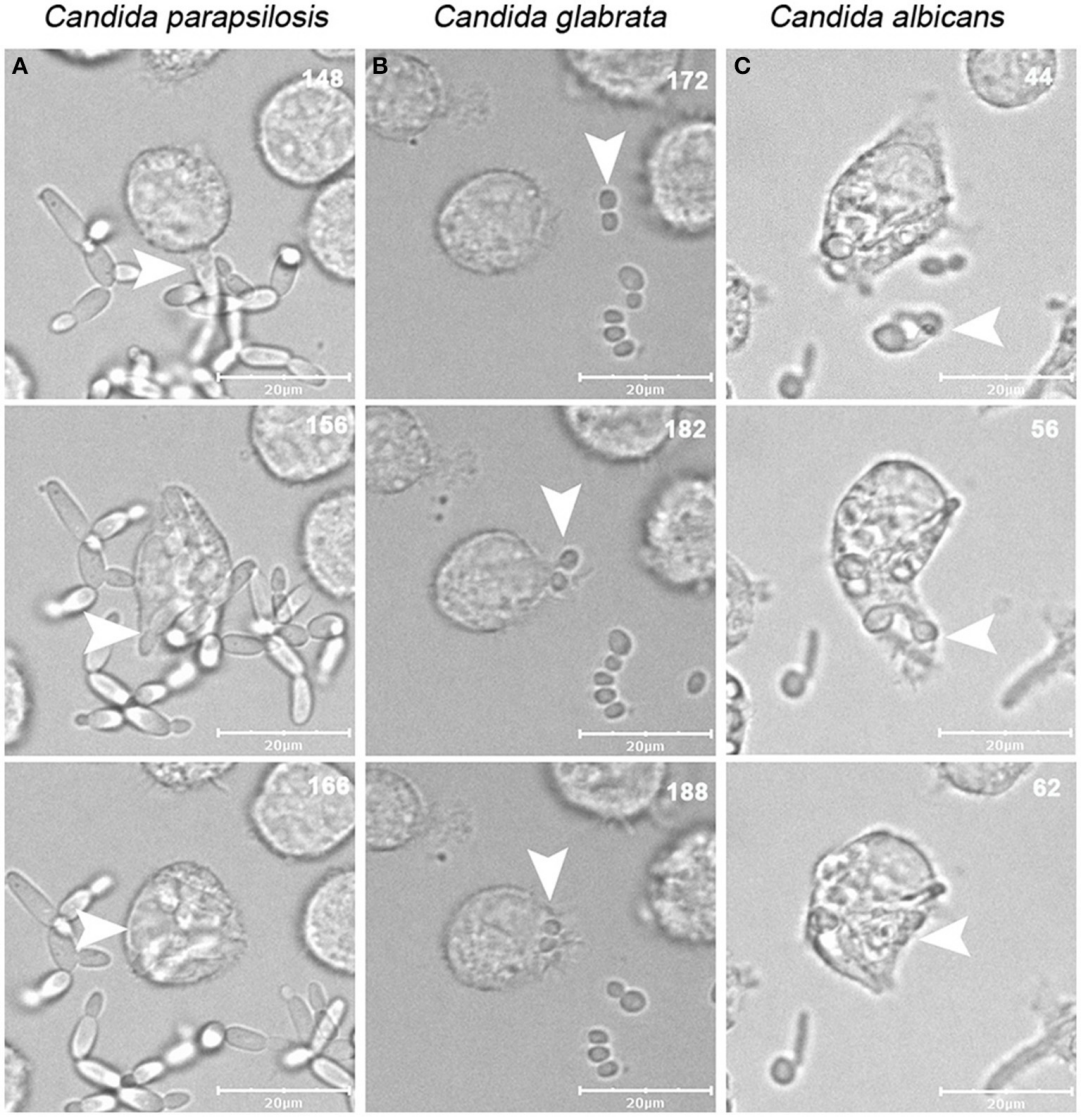
**Uptake of *C. albicans, C. glabrata*, and *C. parapsilosis* strains by J774.1 murine macrophages**. Images were taken from videos made using a spinning-disk confocal microscope during the incubation of phagocytic cells with live *C. albicans, C. glabrata*, and *C. parapsilosis* strains for 6 h on 37°C, in the presence of 5% CO_2_. The numbers in the upper right corner of each image show the time of the phagocytic events, arrows indicate the fungal cells taken up by phagocytic cells over the serial images. Snapshots of J774.1 infection with *C. parapsilosis* **(A)**, *C. glabrata* **(B)**, and *C. albicans* **(C)** are shown. Scale bar: 20 μm.

**Figure 2:**
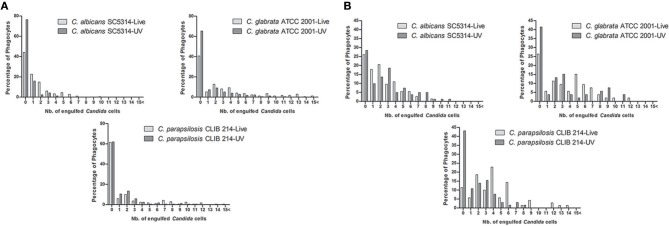
**Distribution of *Candida* cells taken up by macrophages**. Number of live and UV-treated *C. albicans, C. glabrata*, and *C. parapsilosis* cells ingested by J774.1 **(A)** and human PBMC-derived macrophages **(B)** during the 6 h co-incubation period.

**Figure 3:**
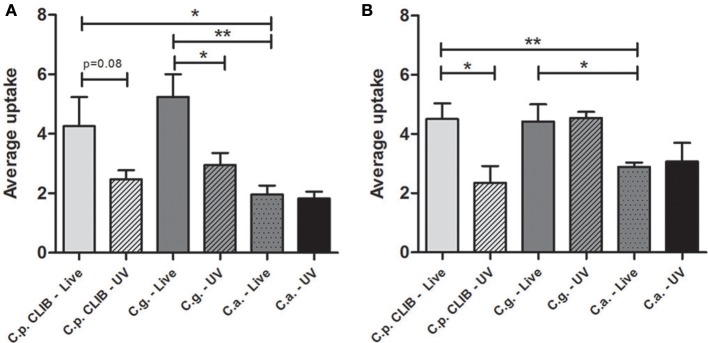
**Average uptake of *Candida* cells by macrophages**. Average uptake of fungal cells by macrophages during the 6 h incubation period after infecting with live or UV-treated *C. albicans, C. glabrata, C. parapsilosis*. **(A)** Murine J774.1 macrophages and **(B)** PBMC-derived human macrophages. Two-tailed *t*-tests were applied, ^*^p < 0.05; ^**^p < 0.01.

Macrophages were infected with live *C*. *albicans, C. glabrata* and *C. parapsilosis* cells at a multiplicity of infection (MOI) of 3 in serum supplemented medium and the phagocytic process was followed by live cell video microscopy. All strains used for this study are well-known clinical isolates (Table 1). Representative images of the engulfment of different *Candida* species by J774.1 macrophages are shown in Figure 1. As expected, differences in phenotype could be observed between the different *Candida* species: while *C. albicans* formed hyphae almost immediately, *C. glabrata* remained strictly in a yeast form, and rapid pseudohypha formation was detectable in *C. parapsilosis*. Here we aimed to compare overall uptake for the different *Candida* species. In general, a greater proportion of the human primary macrophages contributed to uptake of fungal cells than the J774.1 phagocytes. During the 6 h period, a higher percentage of J774.1 macrophages contributed to uptake of *C. albicans* and *C. glabrata* than uptake of *C. parapsilosis* (Figure 2A). In contrast, human PBMC-derived macrophages ingested *C. parapsilosis* cells with higher efficiency compared to the other *Candida* species (Figure 2B). We also found that the number of ingested yeast cells per macrophage differed between species. The majority of the murine and human macrophages contained two *C. albicans* cells; in contrast a greater number of *C. glabrata* and *C. parapsilosis* were phagocytosed by macrophages (Figures 2A,B). Of the macrophages that had taken up at least one fungal cell the average number of phagocytosed *C. albicans* cells was the lowest when applying both J774.1 and human macrophages (mean ± s.e.m., 1.97 ± 0.30 and 2.89 ± 0.15, respectively, Figures 3A,B). However, there was no difference between the mean number of phagocytosed *C. parapsilosis* and *C. glabrata* cells by either J774.1 (4.26 ± 0.98 and 5.24 ± 0.76) or human macrophages (4.51 ± 0.52 and 4.42 ± 0.58 respectively, Figures 3A,B) In addition, the data from J774.1 analysis shows that the average uptake was lower in the presence of the UV killed strains compared to live *C. parapsilosis* and *C. glabrata*, but remained the same for *C. albicans*. Interestingly, these differences did not occur with human PBMC-derived macrophages, although there was a trend toward a lower phagocytic activity against the UV-treated *C. parapsilosis* strain in comparison to live yeast cells.

**Table 1:** **Candida strains utilized for this study**.

**Species**	**Strain**	**References**
*Candida albicans*	SC5314	Gillum et al., 1984
*Candida glabrata*	ATCC 2001	Dujon et al., 2004
*Candida parapsilosis*	CLIB 214	Laffey and Butler, 2005
*Candida parapsilosis*	CLIB 214 *leu2*Δ*::FRT/leu2*Δ*::FRT::cprp10*Δ*::FRT-caRP10::TDH3p-caGFP/cpRP10*	–

### Differences in phagocytosis kinetics between the *Candida* species

**Figure 4:**
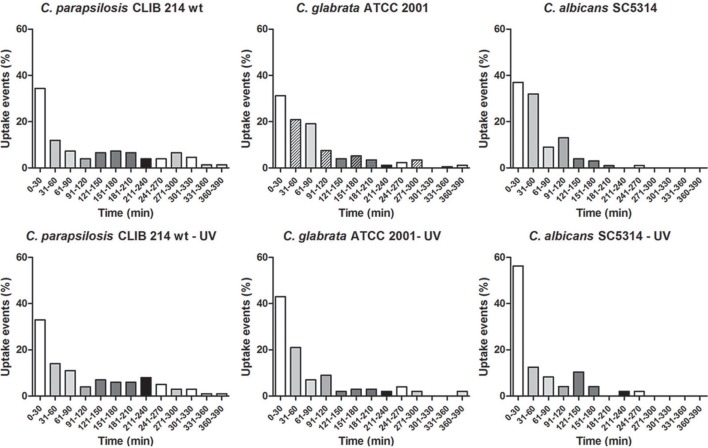
**Prevalence of uptake events by J774.1 macrophages when co-culturing with different *Candida* species**. The percentage of uptake events during the 6 h incubation period of the murine cell line J774.1 with *C. parapsilosis, C. glabrata*, and *C. albicans* live and UV-treated strains. Individual events imply the starting point of the recognition of fungal cells by phagocytic cells.

We also examined whether there are differences in the rates of *C. parapsilosis* uptake in comparison to *C. albicans* and *C. glabrata* by J774.1 macrophages. We found that most of the *C. albicans* cells were taken up during the early phase following infection of macrophages *in vitro* and the number of uptake events decreased with the time, with almost no uptake after 3 h of incubation. In phagocytosis assays with *C. glabrata* and *C. parapsilosis* a similar uptake pattern was observed, however uptake events still occurred after 3 h of the co-incubation. UV-killed strains enhanced uptake of *C. albicans* and *C. glabrata* but did not have any effect on *C. parapsilosis* uptake (Figure 4).

### Macrophage migration toward *C. albicans, C. glabrata*, and *C. parapsilosis*

**Figure 5:**
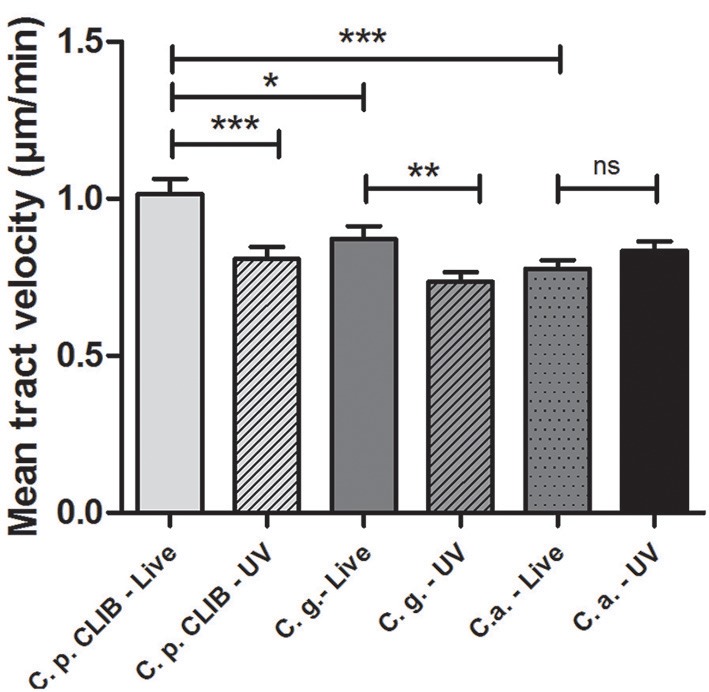
**J774.1 murine macrophage migration toward *Candida* species**. Individual murine J774.1 macrophages were incubated with live or UV-treated *C. albicans* SC5314, *C. glabrata* ATCC 2001, and *C. parapsilosis* CLIB 214 and tracked with the help of Volocity 6.3 software in defined time intervals and measured in μm/s. Diagram shows mean tract velocity values in μm/min. Statistical analysis was carried out using GraphPad Prism applying two-tailed *t*-tests. Significant differences were considered at *p*-values, ^*^p < 0.05; ^**^p < 0.01; ^***^p < 0.001.

We further asked the question whether the differences present in overall uptake are affected by changes in the migration of macrophages toward the species. Therefore we examined the migration of J774.1 murine macrophages in the presence of *C. albicans SC5314, C. glabrata* ATCC 2001, and *C. parapsilosis* CLIB 214. As the greatest phagocytic activity was detected during the early stages of the phagocytosis assay, the movement of macrophages was tracked during the first 45 min. Quantitatively, there were no significant differences between the migration speed of J774.1 macrophages toward *C. albicans* (mean ± s.e.m.; 0.78 ± 0.03 μm/min) and *C. glabrata* (0.87 ± 0.04 μm/min); however, the migration velocity of macrophages in the presence of *C. parapsilosis* was significantly higher (1.02 ± 0.05 μm/min, *p* < 0.05, Figure 5) than in the presence of the other two species. A previous study showed that macrophage migration toward *C. albicans* depends on the cell wall structure of the fungi rather than cell viability (Lewis et al., 2012a). According to our findings, the UV-treatment of fungal cells decreased the mean track velocity of macrophages toward both *C. parapsilosis* (0.81 ± 0.04 μm/min) and *C. glabrata* (0.74 ± 0.03 μm/min) but not *C. albicans* (0.83 ± 0.03 μm/min, Figure 5).

### Differences in engulfment time of *Candida* species by murine and human macrophages

**Figure 6:**
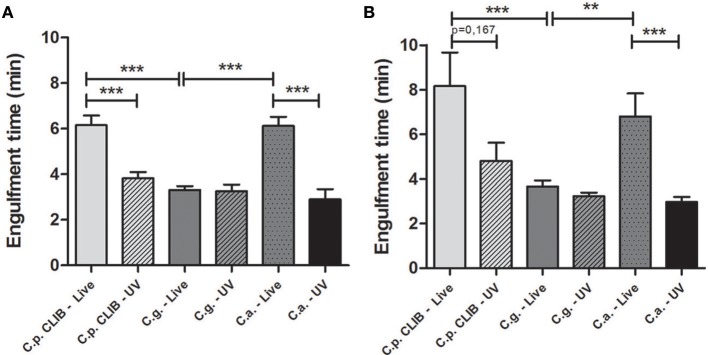
**Engulfment time required for the ingestion of *Candida* cells by J774.1 murine and human PBMC-derived macrophages**. The bars represent the average time (minutes) taken for the complete engulfment of live or UV-treated *C. albicans, C. glabrata*, and *C. parapsilosis* cells by J774.1 **(A)** and human PBMC-derived macrophages **(B)** during the 6 h incubation period. Statistical analysis was carried out using two-tailed *t*-tests. ^**^p < 0.01; ^***^p < 0.001.

The engulfment of *C. albicans* by macrophages has been studied previously (Lewis et al., 2012a). In this study we aimed to assess how engulfment of *C. parapsilosis* differs from *C. albicans* and *C. glabrata* by J774.1 murine and human PBMC-derived macrophages. The engulfment time is defined as the time from first cell-cell contact to complete internalization of the target cell. We found that the engulfment of *C. albicans* and *C. parapsilosis* cells by J774.1 macrophages required significantly more time (mean ± s.e.m., 6.12 ± 0.41 and 6.16 ± 0.42 min, respectively) than that of *C. glabrata* (3.32 ± 0.16 min, *p* < 0.001, Figure 6A). The same pattern with regards to *Candida* spp. engulfment was observed when using human PBMC-derived macrophages, again more time was needed for the uptake of *C. parapsilosis* (8.16 ± 1.51 min) and *C. albicans* cells (6.80 ± 1.04 min) than for the internalization of *C. glabrata* (3.66 ± 0.28 min, Figure 6B). Furthermore, the time required for the engulfment of *C. albicans*, and *C. parapsilosis* but not that of *C*. *glabrata* by J774.1 macrophages decreased significantly following the UV-treatment of yeast cells (Figure 6A). When using PBMC-derived macrophages, we observed a similar trend, although the engulfment time of live *C. parapsilosis* cells was not significantly different from that of UV-killed yeasts (Figure 6B).

### *C. parapsilosis* pseudohypha uptake does not correlate with engulfment time

**Figure 7:**
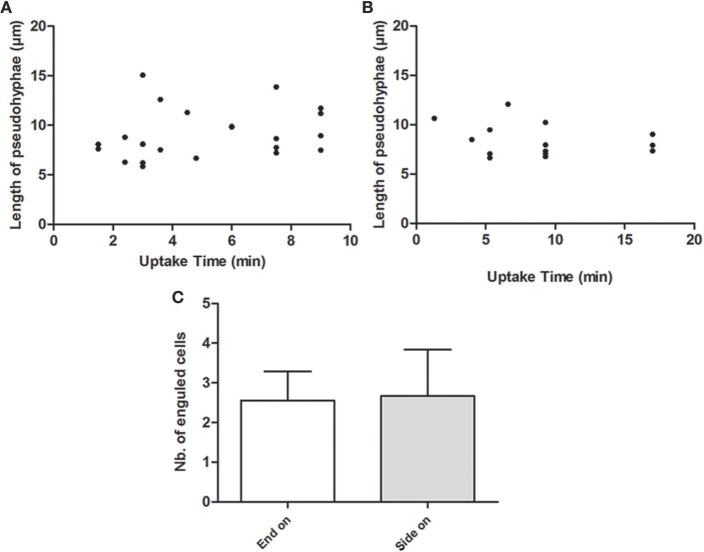
**Role of pseudohyphae length and spatial orientation in the engulfment process**. Figures show the correspondence between the length of pseudohyphae (μm) formed rapidly by *C. parapsilosis* CLIB 214 and the engulfment time (minutes) required for phagocytosis by J774.1 phagocytes **(A)** and by PBMC-DM **(B)**. **(C)** Represents the average number of fungal cells taken up by J774.1 macrophages both from end-on and side-on at which cell-cell contact was initiated.

It has been previously described that for *C. albicans*, the length of hyphae below a 20 μm threshold does not have an influence on engulfment time but their spatial orientation does (Lewis et al., 2012a). Therefore, we addressed the question whether the same applies for the pseudohyphae formed by *C. parapsilosis*. According to our findings, neither the length of pseudohyphae, nor their spatial orientation affected the engulfment time (Figure 7).

### Host cell damage induced by *C. albicans, C. glabrata*, and *C. parapsilosis*

**Figure 8:**
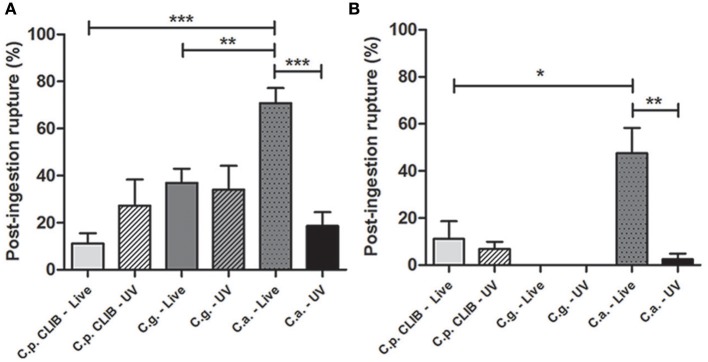
**Post-ingestion rupture of macrophages after co-culturing with different *Candida* species**. Post-ingestion macrophage rupture is defined as the number of bursting macrophages relative to the entire macrophage population after the uptake of live or UV-treated *C. albicans, C. glabrata*, and *C. parapsilosis* during the 6 h incubation period. The percentage of macrophage rupture is shown for J774.1 **(A)** and human PBMC-derived macrophages **(B)**. Two-tailed *t*-tests were applied to determine statistical relevance. ^*^p < 0.05, ^**^p < 0.01; ^***^p < 0.001.

We also compared the capacity of different *Candida* spp. to kill host macrophages. As expected live hyphal *C. albicans* was responsible for the majority of macrophage rupture events after ingestion and UV-killed *C. albicans* caused significantly less damage in case of both types of macrophages (Figures 8A,B). However, there were no major differences in the number of macrophage rupture events caused by live or dead *C. parapsilosis* and *C. glabrata*. Notably, the *Candida* strains caused more damage to J774.1 macrophages compared to human cells, and there were not any rupture events detected following the infection of human macrophages with either live or UV-killed *C. glabrata*.

### Intracellular events after the internalization of *C. parapsilosis* cells

**Figure 9:**
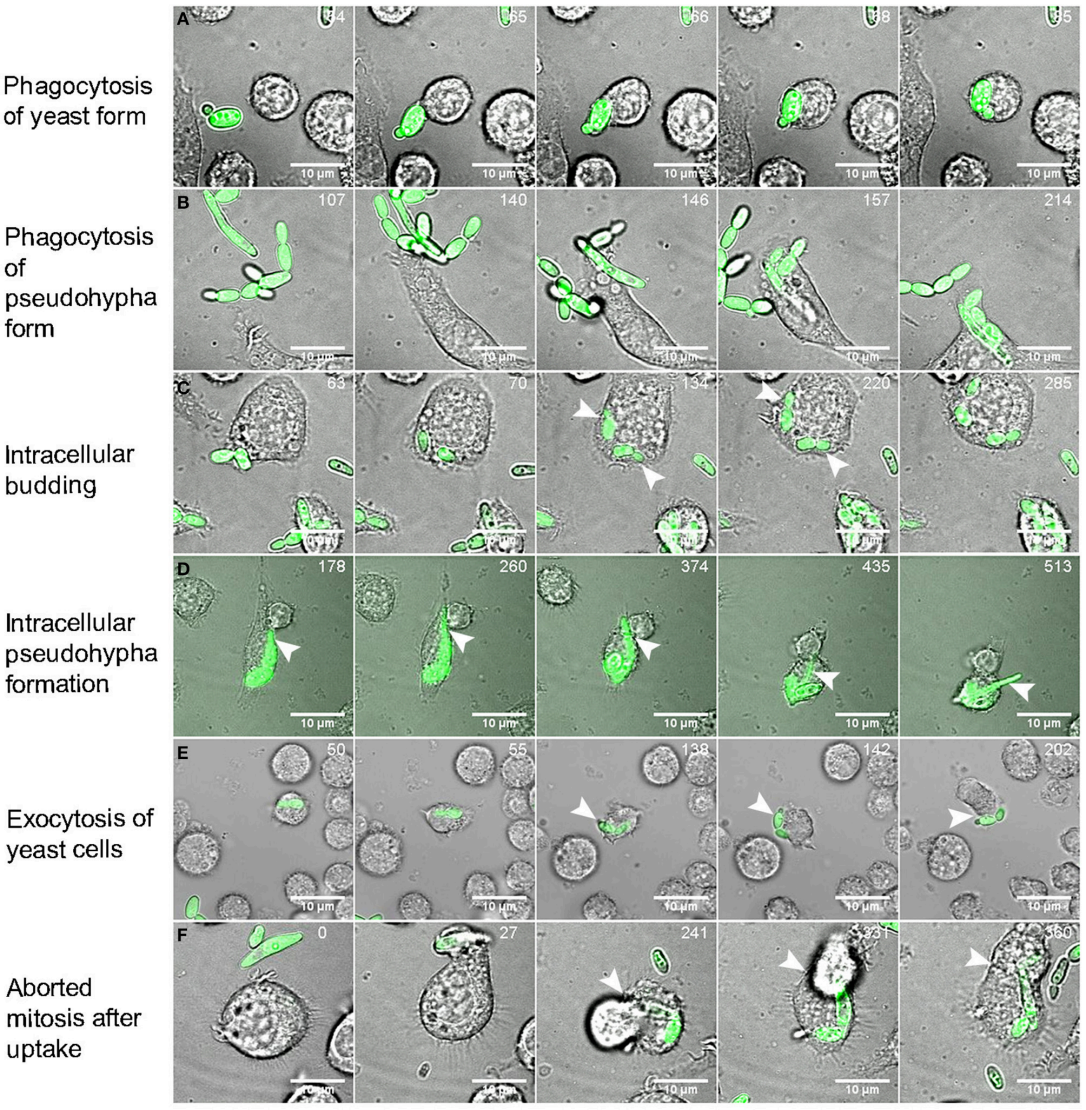
**Intracellular events after the uptake of *C. parapsilosis* cells**. Images were taken from videos made by spinning-disk confocal microscope during the infection of J774.2 murine macrophages with GFP-labeled C. *parapsilosis* cells. Numbers at the upper right corner of the pictures show the duration of time (minutes) when observing certain events. Snapshots show the phagocytosis of yeast forms **(A)** and pseudohyphae forms **(B)** of *C. parapsilosis*, intracellular replication **(C)** with the newly formed buds (arrows) and intracellular pseudohyphae formation shown by arrows **(D)**. Exocytic mechanism was also captured shown on snapshot series **(E)**. Arrows indicate the exocytosis of a fungal cell after its uptake. The event of aborted mitosis is shown in **(F)**. After the engulfment of *C. parapsilosis* cells the phagocytic cell start to divide (241 min), but instead of separating, the two attached daughter cells fuse leading to aborted mitosis (360 min). Scale bar: 10 μm.

Previous studies have demonstrated how *C. albicans* and *C. glabrata* respond to the restrictive environment of phagosomes in order to survive (Benjamin et al., 2004; Seider et al., 2011; Brunke and Hube, 2013; Vylkova and Lorenz, 2014). However, the survival strategies of *C. parapsilosis* have not been investigated to date; therefore, we analyzed the intracellular events following phagocytosis. A representative 3D video of the interaction between the J774.1 macrophages and *C. parapsilosis* cells is shown in Video S1. For the infection of the J774.1 macrophages, GFP labeled *C. parapsilosis* (Figure 9, Videos S2–S7) and unlabeled *C. parapsilosis* (Video S8) isolates were used. The following intracellular events are available to view in Videos S2–S8. When studying the post engulfment events of both *C. parapsilosis* morphological forms (Figures 9A,B, Videos S2–S3), we observed intracellular budding (Figure 9C, Video S4) and pseudohyphae formation (Figure 9D, Video S5). Non-lytic expulsion of *C. albicans* by J774.1 macrophages has already been shown (Bain et al., 2012). Infrequently, we were also able to observe the exocytosis of *C. parapsilosis* cells by murine macrophages (Figure 9E, Video S6).

Aborted mitosis of the host cell after the uptake of *C. albicans* has been reported previously (Lewis et al., 2012b; Schafer et al., 2014). After the engulfment of fungal cells, the phagocytic cell starts to divide but instead of separating, the two attached daughter cells fuse back together. This event is more frequent when intracellular hyphae forming occurs (Lewis et al., 2012b). Interestingly, we found the same event after the uptake of *C. parapsilosis* even though this species is able to form only pseudohyphae instead of true hyphae (Figure 9F, Video S7). The previously presented individual events could also happen at the same time followed by post-ingestion rupture of macrophages as shown in Video S8.

## Discussion

Previous studies on phagocytosis kinetics related to *Candida* species have focused mainly on *C. albicans*, and little information is available about the phagocytosis of non-albicans *Candida* species, especially that of *C. parapsilosis*. Here we investigated the physical interaction between the cellular components of the innate immune system and different *Candida* species by studying separate stages of the phagocytosis process. We compared the engulfment and recognition of a *C. parapsilosis* clinical isolate to *C. albicans* and to another *Candida* species, *C. glabrata* by using both murine J774.1 and human PBMC-derived macrophages. Besides visualizing host-pathogen interactions, live cell imaging enabled us to quantitatively analyze the phagocytic process. Kinetic studies allowed for the calculation of the engulfment time required for the internalization of fungal cells, macrophage migration speed toward *Candida* species, along with determining the overall uptake of the different species and post-ingestion rupture of macrophages. When monitoring the movement of J774.1 murine macrophages, we detected significant differences as macrophages showed increased track velocity toward *C. parapsilosis* cells in comparison to *C. albicans* and *C. glabrata*. The UV-inactivation of all strains decreased the migration speed of macrophages toward both *C. parapsilosis* and *C. glabrata* but not *C. albicans*.

Even though the mean tract velocity of macrophages was the highest toward *C. parapsilosis*, the clearance of yeast cells prolonged in time, and a lower number of murine phagocytes contributed to their uptake compared to C. *albicans* and *C. glabrata*. Interestingly the opposite was observed with human primary cells: more phagocytes ingested *C. parapsilosis* cells compared to the other two species. Although both murine and human phagocytes needed a relatively long time to engulf individual *C. parapsilosis* cells, multiple number of yeast cells were taken up. In spite of the great amount of fungal cells ingested, the number of post-ingestion macrophage rupture events remained low. The UV-treatment of *C. parapsilosis* cells decreased the engulfment time along with the average uptake by both types of macrophages. Also significant decrease in the speed of murine macrophages was detected following the UV-treatment of *C. parapsilosis* cells. However, no changes occurred neither in phagocytosis kinetics, nor in the rate of macrophage killing in comparison to live cells.

*C. albicans* cells have been reported to form hyphae rapidly after their internalization by macrophages (Benjamin et al., 2004). Although the macrophage migration toward *C. albicans* was significantly slower than toward *C. parapsilosis*, the clearance of *C. albicans* was faster during the entire co-incubation period by murine macrophages. Furthermore, more J774.1 phagocytes were actively ingesting this species than *C. parapsilosis*, however, as mentioned above, the opposite was observed in the case of human primary macrophages. The engulfment time of *C. albicans* was as high as that of *C. parapsilosis*, however only a small amount of *C. albicans* cells were ingested by both types of macrophages. In most cases, *C. albicans* was responsible for the majority of the post-ingestion macrophage rupture events, indicating that it is the most virulent among the three species. As it has been previously observed (Mckenzie et al., 2010) host cell damage occurred mainly due to rapid hypha formation of *C. albicans* after the uptake, rather than because of the ingestion of large number of yeast cells. UV-treatment of *C. albicans* cells did not affect the macrophage migration speed, however significantly decreased host cell damage that is in line with the findings of others (Lo et al., 1997; Mckenzie et al., 2010). Also UV-killing of *C. albicans* cells resulted in enhanced clearance by murine macrophages. The engulfment time of UV-killed *C. albicans* shortened, although the rate of average uptake remained the same compared to live cells when using either murine or human phagocytes. Furthermore as expected, UV-killing of *C. albicans* cells significantly decreased the prevalence of post-ingestion macrophage rupture events due to lack of hypha formation (Lo et al., 1997; Mckenzie et al., 2010).

Even though the speed of macrophages was lower toward *C. glabrata* than toward *C. parapsilosis*, the clearance of *C. glabrata* cells also prolonged in time as well as in the presence of *C. parapsilosis* cells, leading to uptake events even after 3 h of the co-incubation with murine phagocytes. When comparing the actively ingesting macrophages the same tendency was detectable as observed in case of *C. albicans*: more murine macrophages were phagocytosing *C. glabrata* cells (similarly to the rate of *C. albicans*) than *C. parapsilosis* cells, however the opposite was shown when using human phagocytes. According to the overall uptake data, both types of macrophages ingested multiple number of *C. glabrata* cells similarly to *C. parapsilosis*, however the engulfment time was significantly shorter. *C. glabrata* is the only human pathogenic *Candida* species that is not able to switch from a yeast form to any other forms above the temperature of 37°C, although one report has described pseudohypha formation at an extremely infrequent rate (Fidel et al., 1999; Csank and Haynes, 2000; Benjamin et al., 2004). In contrast, *C. parapsilosis* has the ability to undergo morphological changes and produce pseudohyphae (Trofa et al., 2008). According to our findings while the formation of secondary structures increases the engulfment time, the UV-killing of cells caused inability to change morphology therefore shortens the engulfment rate.

The rate of post-ingestion macrophage rupture events due to *C. glabrata* remained low similarly to *C. parapsilosis*, in addition no rupture events were detected when using human primary cells. This finding indicates that in regard to phagocytosis, *C. glabrata* is the least virulent among all three *Candida* species. Reports have demonstrated the survival of *C. glabrata* cells in immunocompetent mice even weeks after the infection and also survival was reported in phagocytic cells by neutralizing the phagosome (Seider et al., 2011). The data based on the post-ingestion rupture of macrophages also supports these findings for the persistence of *C. glabrata*. UV-treatment of *C. glabrata* cells decreased the macrophage migration velocity, although there was a tendency toward a slightly faster clearance by J774.1. In case of both types of macrophages the engulfment time remained the same as when applying live cells, however while murine macrophages ingested less UV-killed *C. glabrata* than live cells, the average uptake of both live and UV- killed *C. glabrata* by human primary phagocytes remained the same.

Furthermore we have shown that the rate of actively phagocytosing macrophages was lower in the case of J774.1 macrophages and *Candida* strains caused more damage to these macrophages compared to the human primary cells. Previously we have reported that J774.1 and primary human macrophages do not have the same activity against *C. parapsilosis*: while human macrophages kill *Candida metapsilosis* and *Candida orthopsilosis* with higher efficiency compared to *C. parapsilosis sensu stricto*, J774 cells do not differentiate between the three species (Nemeth et al., 2013). Although the reason of these differences is unknown, we may speculate that the differential expression of pattern recognition receptors on the surface of phagocytes might be responsible for these findings. As a conclusion, the data gained from the experiments with cell lines and primary cells at the same time should be treated with increased attention as certain differences are more evident once applying primary cells while others become irrelevant.

For *C. albicans*, reports have confirmed that engulfment of hyphae under 20 μm does not influence the engulfment rate of macrophages however their spatial orientation does (Lewis et al., 2012a). Even though *C. parapsilosis* CLIB 214 cells rapidly form pseudohyphae, neither their length under 20 μm, nor their spatial orientation influenced the speed of internalization by J774.1 macrophages. Thus while pseudohyphae are considered to be potential virulence factors contributing to biofilm formation and host tissue penetration (Trofa et al., 2008) their presence does not seem to have a major influence on phagocytosis. This finding could suggest that the hyphal and pseudohyphal forms of certain species are recognized in different ways by these macrophages.

During these experiments we have seen various outcomes when examining the distinct stages of the phagocytic process: migration, engulfment and killing of host cell. Multiple factors can influence the different stages. For example, formation of secondary structures can influence the mechanism of uptake, also macrophage migration toward the species can be effected by either the presence or the lack of certain fungal signaling molecules, possible differences in the cell wall composition could have an effect on the engulfment time and uptake kinetics. However, further investigations are required to reveal these species specific differences.

In some cases, the velocity of macrophages was increased toward one species along with the overall uptake. Similarly when the speed of macrophages decreased, the rate of overall uptake decreased as well. This suggests a certain type of connection between the two stages. Although in other cases, while the macrophage velocity was low, the overall uptake was high.

In addition, in this study we have seen that even though murine and human phagocytes ingested multiple numbers of *C. parapsilosis* and *C. glabrata* cells, phagocytosed *C. albicans* seemed to cause most of the damage. As expected, this was mainly dependent on the morphological changes of *C. albicans* cells, rather than the number of ingested yeast cell leading to the conclusion that there is no strong correlation between the number of ingested cells and host cell damage. Taken together all these findings drive us to the conclusion that the distinct stages of the phagocytic process are not closely and necessarily related to each other. Therefore studying the individual stages of the phagocytic process instead of examining it in its entirety could be more informative when investigating host-pathogen interactions.

Opportunistic fungi have developed multiple immune evasion strategies to survive within phagocytic cells after their uptake. Several strategies are used commonly: piercing or escape from macrophages; silencing or avoiding lysis transiently by the inhibition of phagolysosome fusion, or surviving in the acidic environment inside macrophages, and also triggering pyroptotic cell death of infected macrophages (Hummert et al., 2010; Wellington et al., 2012, 2014). As a result, the engulfed fungal cells might be able to replicate inside macrophage phagosomes. Intracellular replication has been reported with *C. albicans* and *C. glabrata*, however not when studying *C. parapsilosis* (Jong et al., 2001; Roetzer et al., 2010; Seider et al., 2011, 2014). Here we highlight that *C. parapsilosis* has the ability not only to survive during restricted environmental conditions but to replicate and form pseudohyphae after ingestion by macrophages over a 6 h co-incubation period. The videos taken by spinning disk confocal microscopy verify these events including intracellular budding, pseudohyphae growth, exocytosis of yeast cells and aborted mitosis of phagocytic cells after the infection of J774.1 macrophages with *C. parapsilosis* cells.

Although *C. albicans, C. glabrata and C. parapsilosis* are closely related pathogens, they induce different host responses during an infection and use different survival strategies in order to survive in a patient. Here we have demonstrated how phagocytosis and the clearance of *C*. *parapsilosis* vary at several points from other *Candida* species and that this organism also has the ability to survive under restrictive environmental conditions such as the acidic compartment of macrophage phagosomes.

### Conflict of interest statement

The authors declare that the research was conducted in the absence of any commercial or financial relationships that could be construed as a potential conflict of interest.
