# Supplementary material for: Kinetic studies of Candida parapsilosis phagocytosis by macrophages and detection of intracellular survival mechanisms
Source: Front Microbiol. 2014 Nov 20;5:633. doi: 10.3389/fmicb.2014.00633 (PMC4238376; doi:10.3389/fmicb.2014.00633)
Supplement: Supplementary file 9 [file DataSheet1.DOCX]

**Supporting Videos**

**Video S1. 3D Projection of *Candida parapsilosis* and murine macrophage interaction.** Showing a 3D snapshot of FITC - stained *Candida parapsilosis* yeast cell uptake by a J774.1 macrophage. For labeling the acidic compartments of the murine phagocyte lysotracker red DND-99 was applied. (WMV file)

**Video S2. Live Cell Imaging of Yeast cell phagocytosis.** Shows an example of a *C. parapsilosis* yeast cell phagocytosis by a murine macrophage. GFP – tagged *C. parapsilosis* CLIB 214 cells were used for the infection. (AVI file)

**Video S3. Live Cell Imaging of Pseudohyphae Phagocytosis.** The representative video shows a murine macrophage internalizing a GFP – tagged *C. parapsilosis* pseudohypha. (AVI file)

**Video S4. Live Cell Imaging of Intracellular Budding.** Shows live GFP – tagged *C. parapsilosis* cells being ingested by J774.1 macrophages. After the uptake of fungal cells budding occurs and newly formed daughter cells are visible inside of the phagocytes. (AVI file)

**Video S5. Live Cell Imaging of Intracellular Pseudohypha growth.** Shows pseudohypha growth of an internalized GFP - tagged *C. parapsilosis* cell. The video further shows a failed attempt of the macrophage to fold the constantly growing elongated structure which results in the rupture of the host cell (AVI file).

**Video S6. Live Cell Imaging of Exocytosis.** Respresentative video shows the exocytosis of previously internalized GFP - tagged *C. parapsilosis* cells. During the process yeast cell budding is again observable. (AVI file)

**Video S7.** **Live Cell Imaging of Aborted Mitosis**. Shows aborted mitosis of the murine host cell after the internalization of GFP-tagged *C. parapilosis* cells. Following the uptake of fungal cells, the host cell starts to divide but instead of separating, the two attached daughter cells fuse back together. (AVI file)

**Video S8. Live Cell Imaging of Combined Intracellular events**. Shows combined intracellular events after the engulfment of *C. parapsilosis* yeast cells: aborted mitosis of the host cell, intracellular pseudohyphae forming followed by macrophage rupture. Acidic compartments of the murine cells were labeled with lysotracker red. (AVI file)
